# Knowledge, attitude and practice towards COVID-19 among workers of a tertiary hospital in Bayelsa State, Nigeria

**DOI:** 10.11604/pamj.supp.2020.37.1.26259

**Published:** 2020-10-14

**Authors:** Oghenekaro Godwin Egbi, Chika Duru, Benedicta Kasia

**Affiliations:** 1 Department of Internal Medicine, College of Health Sciences, Niger Delta University, Amassoma, Bayelsa State, Nigeria,; 2 Department of Pediatrics, College of Health Sciences, Niger Delta University, Amassoma, Bayelsa State, Nigeria,; 3 Department of Chemical Pathology, College of Health Sciences, Niger Delta University, Amassoma, Bayelsa State, Nigeria

**Keywords:** Attitude, knowledge, coronavirus disease, hospital staff, health workers, pandemic, practice

## Abstract

**Introduction:**

coronavirus disease (COVID-19) has since assumed pandemic dimensions with over 14million persons affected in 213 countries and territories worldwide as at mid-July 2020. The level of awareness and knowledge of the disease as well as the related attitude and practice amongst hospital workers could determine its extent of control. The aim of this study was to determine the knowledge, attitude and practices amongst hospital staff regarding COVID-19.

**Methods:**

this cross-sectional study was conducted among staff of the Niger Delta University Teaching Hospital, Bayelsa State, Nigeria. A self-administered semi-structured questionnaire was adapted in assessing their knowledge, attitude and practice towards COVID-19.

**Results:**

one hundred and twenty four respondents completed the study with a mean age of 30.9 ± 6.5 years. Up to 90% of them demonstrated good knowledge of COVID-19 with regard to preventive and control measures. Approximately 90% of them practiced good hand hygiene though a lesser proportion wore face masks (51.6%) and practiced crowd avoidance (58.1%). Medical doctors had a marginally higher practice score compared with “others” (p = 0,047). Up to 98(79.0%) of respondents were scared of self-infection and 99(79.8%) were not motivated to work due to inadequate facilities, equipment and resources in 50% of cases. Knowledge of COVID-19 was positively correlated with the practices of the participants (p = 0.023).

**Conclusion:**

the hospital staff lacked adequate motivation towards management of COVID-19 and were constrained for the fear of self- infection, inadequate equipment, resources and equipment despite possessing a good knowledge of the disease. There is need for concerted efforts by stakeholders in the health care industry to ensure hospital workers are adequately motivated and provided with the needed risk protection devices and resources during this pandemic.

## Introduction

Coronavirus disease 2019 (COVID-19) is a new and highly infectious respiratory disease caused by a novel coronavirus and was first detected in December 2019 in Wuhan, China [1]. From China, the COVID-19 epidemic continued to spread to involve progressively greater number of counties and continents globally. On the 30^th^ of January, 2020 it was declared a global emergency of international concern [2] and by 11^th^ of March 2020, the World Health Organization (WHO) declared it a pandemic and called for collaborative efforts by all countries to prevent its rapid spread [3]. As at the time of this report in July 2020, up to 14 million persons had been affected in 213 countries and territories globally. Nigeria recorded its first case on the 28^th^ of February 2020 from an Italian returnee. Since then it has joined other members of the African continent as one of the countries seriously hit by the epidemic. Although the mortality rate of the disease remains quite low, put at less than 5% [4], it is highly infectious and transmissible and has no cure as at yet. To prevent widespread community transmission of COVID-19, some unprecedented global measures were adopted such as closure of interstate air and land borders, schools, worship centers and market places and identification and isolation of suspected and confirmed cases through case detection and contact tracing. Other measures included advice to residents to stay at home, compulsory wearing of face masks in public, frequent hand washing and use of hand sanitizers as well as physical distancing [5]. However, despite these precautionary measures being observed in several places, the battle against COVID-19 continues to rage on with an exponential increase in confirmed cases in many quarters. Adherence to these preventive and control measures is therefore crucial at all levels to help curb this scourge. Previous studies have shown that the knowledge of a disease and its treatment options improves adherence [6]. A hospital environment consists of health care workers (HCWs) as well as other employees who work within the hospital community. Protection of hospital workers and prevention of nosocomial transmission of infection are crucial in the epidemic response necessitating that health workers have updated knowledge about the source, transmission, symptoms and preventive measures of the disease [7]. Poor knowledge may result in delayed diagnosis, spread of disease and poor infection control practices [8]. The level of preparedness of health workers to manage epidemics may affect the extent to which the disease is curtailed. There is need to determine their level of awareness and practice related to COVID-19. Since COVID-19 is an emerging disease, there is limited data on this subject, especially in developing countries. The aim of this study was therefore to assess the knowledge, attitude and practices towards COVID-19 among staff of the Niger Delta University Teaching Hospital (NDUTH), a designated isolation centre in Bayelsa State, Nigeria during the rapid rise period of the outbreak in Nigeria.

## Methods

**Study site:** this descriptive cross-sectional survey was carried out among health workers of NDUTH from April to July 2020. NDUTH is a tertiary health facility located in Okolobiri, a semi-urban town in Bayelsa State, South- South Nigeria. It serves as the teaching hospital to the Niger Delta University which is located about 20 kilometers away. The hospital has a total of about 539 staff of varying cadres working in different departments. This includes doctors, nurses, nursing assistants, medical laboratory scientists and technicians, pharmacists, physiotherapists, radiographers and dieticians. Others include the medical records officers, administrative staff, and janitors. The hospital also serves as a residency training centre and hosts internship programs for doctors, nurses, pharmacists and medical laboratory scientists. The NDUTH is one of the two tertiary centres in Bayelsa State and is the foremost designated referral isolation centre for the management of patients with COVID-19 in the state. At the onset of the study in early April, Bayelsa state was yet to record a case of COVID-19 though there were already cases in surrounding states. However as the study progressed towards mid-July, over 300 confirmed cases of COVID-19 had been recorded with about 20 fatalities.

**Study population:** all available health care professionals and other workers of NDUTH were selected through convenient sampling for this survey which assessed their knowledge, attitude and practice towards COVID-19. For the purpose of the study, the hospital workers were divided into three categories: doctors, nurses/nursing assistants and “others.” The “others” category included paramedics (medical laboratory scientists, pharmacists, radiotherapists, dieticians), hospital administrative staff as well as janitors.

**Conduct of the study:** a self-administered semi-structured questionnaire was used to collect data from the participants. The questionnaires were distributed by two medical interns who had been previously trained by the authors. In accordance to the rules on physical distancing, they maintained a minimum distance of 1m away from respondent and each other. They also wore gloves which were disposed of and replaced after attending to each participant. Each interviewer and participant also had access to hand sanitizers.

**Study instrument:** the questionnaire had two main parts. The first part contained questions on participants´ demographics such as age, gender, marital status, educational attainment, job category (doctors, nurses / nursing assistants, and others), as well as work experience (years). The second part of the survey contained items assessing participants´ knowledge, attitude and practice (KAP) concerning COVID-19. The KAP questions were adapted from a validated questionnaire used in previous studies [9]. The score of each individual item was summed up to give a composite score for each category. The knowledge section comprised of 14 items made up of questions regarding etiology and risk of disease (2- items), disease transmission (2-items), clinical features (2-items), treatment (2-items) and precautions/preventions (5-items) as well as source(s) of knowledge of COVID-19. Each of the first 13 question was responded as “yes”, “no” and “I don´t know”. The correct answer was marked as 1 while a “no”, “don´t know” or unanswered question earned “0” point. Total score ranged from 0-13. Responses on the last question on source(s) of knowledge of COVID-19 (with multiple options allowed) was noted but not allotted any score. The attitude section comprised of items assessing attitude of hospital staff toward treatment and infection control procedure for COVID-19. Attitude was assessed through four closed-ended questions regarding respondent´s level of fear of contracting coronavirus infection while rendering professional service and confidence in defeating the virus. Response of each item was recorded as either a “yes” or “no”. There were also two follow up open- ended sub-questions that evaluated participants´ initial response to the earlier closed- ended questions. Participants were asked to give reasons for their motivation or ´lack of motivation´ in care of COVID-19 patients whichever was applicable. Participants were also asked to state why they felt or otherwise that Nigeria could win the battle against the disease. Practices were measured through seven preventive or precautionary items including the avoidance of crowd, frequency of hand washing, use of sanitizers, proper use of personal protective equipment (PPE) as well as participation in training on COVID-19. Each item was responded as yes (1-point), no (0-point), or unanswered (0-point) with total score ranging from 0-7.

**Ethical consideration:** the study was approved by the Research and Ethics Committee of NDUTH. Informed consent was obtained from all participants after due explanation of the nature, purpose and objectives of the study. The principles of voluntary participation, privacy, confidentiality and anonymity of subjects were considered and adhered to.

**Statistical analysis:** data was analyzed using IBM SPSS version 20.0. Descriptive analysis was done for continuous variables using measures of dispersion such as mean, standard deviation and range. Discrete variables were presented as frequencies and percentages. The independent t test was used to compare the means of two groups but where more than two groups were involved. ANOVA was used followed by post hoc analysis (Turkey HSD) for significant variables. Data was presented in tabular and graphical forms. For all analyses, two-tailed P<0.05 was indicative of statistical significance.

## Results

**Socio-demographic characteristics of participants:** one hundred and twenty-four participants completed the study out of the initial 125 workers selected for the study. Only one respondent did not complete it giving a non-response rate of 0.8%. There were 38(30.6%) medical doctors, 39(31.5%) nurses and nursing assistants and 47(37.9%) “others”. They were relatively young with a mean age of participants of 30.9± 6.5 years (range of 19-54 years) and a mean work experience of 2.96 ± 2.61 years and ranging from 0 to 12 years. There were 64 females, comprising 54.8% of the total. Sixty-nine of the participants were “never married” while 55 were “ever married”. Almost all the participants (98.4%) were Christians. Majority (73.4%) of the participants had a graduate degree, 17(13.7%) had a post-graduate degree while 16(12.9%) had a secondary level of education (Table 1).

**Table 1:** socio-demographic variables with corresponding mean KAP scores in the participants

	N	Knowledge Score Mean (SD)	t/F (p)	Attitude Score Mean (SD)	t/F (p)	Practice Score Mean (SD)	t /F (p)
**Age (yrs)**	124		3.036 (0.003)^**^		-0.538 (0.592)		0.638 (0.525)
<30	71	10.79(1.393)		1.46(1.263)		4.63(1.606)	
>30	53	10.00(1.481)		1.58(1.184)		4.44(1.697)	
**Gender**	124		0.086 (0.931)		0.601 (0.549)		-0.045 (0.964)
Males	56	10.46(1.595)		1.59(1.156)		4.55(1.271)	
Females	68	10.44(1.386)		1.46(1.286)		4.56(1.277)	
**Marital Status**	124		2.087^*^ (0.039)		0.645 (0.520)		0.929 (0.355)
Never Married	69	10.70(1.565)		1.54(1.239)		4.68(1.597)	
Ever Married	55	10.15(1.311)		1.45(1.214)		4.40(1.695)	
**Job Category**	124		7.397 (0.001)^**^		9.742 (0.000)^**^		2.682 (0.056)
Medical Doctors	38	10.82(1.335)		1.82(1.023)		4.97(1.460)	
Nurses and NA	39	10.84(1.175)		1.92(1.323)		4.63(1.246)	
Others	47	9.83(1.619)		0.94(1.092)		4.13(1.400)	
**Work Experience**	124		1.367 (0.174)		3.020 (0.085)		0.045 (0.833)
< 2 yrs	71	11.04(1.408)		1.35(1.135)		4.59(1.609)	
> 2yrs	53	10.69(1.490)		1.74(1.318)		4.53(1.694)	
**Educational status**	124		5.750 (0.004)^**^		2.259 (0.109)		19.463 (0.000)^**^
At most secondary	16	9.38(1.500)		1.47(1.187)		2.40(1.682)	
Graduate	91	10.67(1.342)		1.38(1.272)		4.82(1.382)	
Postgraduate	17	10.29(1.759)		2.08(0.827)		5.12(1.536)	

KAP = knowledge, attitude, practice NA= Nursing Assistants, yrs = years ^**^statistically significant

**Knowledge of the participants towards COVID-19:** the average overall knowledge score for all participants was 10.5 ± 1.5 with a range of 6.0 - 13.0. There was a statistically significant difference in knowledge scores between the three categories of hospital workers. (p = 0.001) (Table 1). Nurses / nursing assistants and doctors had significantly higher mean scores than the “others” with a significant post hoc analysis (turkey HSD) p of 0.004 each. Similarly, participants who were younger than 30 years had a higher mean score than those who were older (p= 0.003). Individuals who were “never married” also had a higher knowledge score than their ´ever married´ counterparts (p = 0.039) (Table 1). Graduates had higher mean knowledge score than those with a secondary education (ANOVA post hoc p = 0.03). However, there was no mean difference in knowledge scores between those who had post-graduate and those with graduate level of education. (p = 0.578) or post-graduate and those with secondary level of education (0.157). There was no statistical difference in mean knowledge scores across gender or work experience (P >0.05). Over 96% of the participants knew about the role of isolation in COVID-19 while 92% knew about the importance of supportive care despite the absence of a cure While about 97% of respondents knew about the effectiveness of handwashing and hand sanitizers in helping to prevent spread, 74% of them knew about the importance of face masks. While almost 90% knew about the main symptomatology of COVID-19 and could identify respiratory droplets as the main source of infection, a smaller proportion knew about details of transmission. Up to 70% of respondents answered wrongly that individuals without a fever could not transmit the infection and 30% thought that the infection could be transmitted by eating wild animals (Table 2). The major sources of knowledge of COVID-19 among the participants were social media (42.7%) and television / radio programs (30.6%). Other sources included hospital seminars (25.8%), colleagues or friends (17.7%) and journal or newspapers (11.3%).

**Table 2:** participants’ responses to questions bordering on knowledge of COVID-19

Knowledge item	Proportion n (%)
Eating or contacting wild animals would result in infection by COVID-19 (W))	38(30.6)_
Main symptoms of COVID-19 are fever, fatigue, dry cough and myalgia (C)	110(88.7)
Covid-19 spreads via respiratory droplets of infected individuals (C)	111(89.5)
Persons with COVID-19 infection cannot transmit the virus if fever is absent (W)	87(70.2)
Unlike common cold, stuffy nose, running nose and sneezing are less common in COVID-19 (C)	78(62.9)
Not all patients with COVID-19 will progress to severe cases but the elderly, obese and those with chronic illness are more likely (C)	97(78.2)
Isolation and treatment of individuals infected with COVID-19 are effective ways to reduce spread (C)	121(97.6)
No effective cure for COVID-19 but early, supportive care can help most patients recover (C)	114(91.9)
Regular hand washing and use of sanitizers can effectively help to prevent the spread of COVID-19 (C)	120(96.8)
Individuals can wear general medical masks to reduce chances of spread of the infection (C)	92(74.2)
To prevent infection, individuals should avoid crowded places (C)	118(95.2)
Contacts of someone infected with COVID-19 should be isolated and observed for 14 days (C)	120(96.8)
It is not necessary for children and young adults to take measures to prevent infection by COVID 19 (W)	90(72.6)

C= correct response, W= wrong response

**Figure 1:**
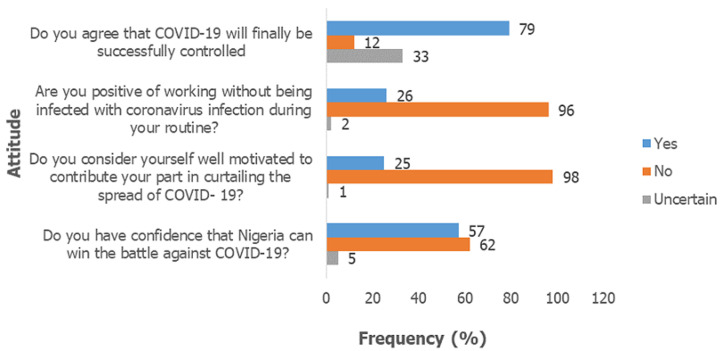
attitude of hospital workers towards COVID-19

**Figure 2:**
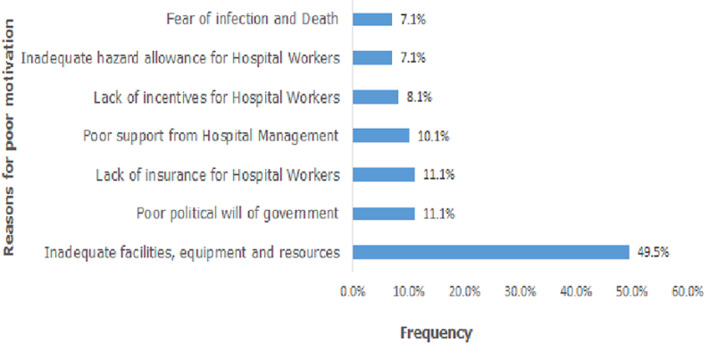
reasons for poor motivation among hospital workers for control of COVID-19

**Attitude of the participants towards COVID-19:** the attitude of the participants towards COVID-19 is represented in Figure 1. Seventy nine (63.7%) participants believed that COVID-19 will finally be successfully controlled. Fifty-seven (46.0%) participants expressed confidence that Nigeria will be able to win the battle against the disease. The commonest reasons ascribed to this confidence was belief in divine intervention in 26.3%, synergistic effect of stakeholders in 21.0% and political will of government (19.3%). However, a greater proportion of respondents did not think Nigeria will be able to successfully combat the disease. Reasons adduced to this included poor government political will, corruption and greed in majority of cases (64.2%). Others were poor funding of health care facilities and inadequate health measures (14.9%) as well as poor education and mobilization of the masses (6.5%). Only 26 (20.1%) respondents were positively working without the fear of infection by COVID-19. Thirteen (12.5%) individuals reported that they were “extremely scared” of becoming infected while another 38 (30.6%) reported that they were “very scared” of the infection. Twenty two (17.7%) were “moderately scared” while 23 (18.5%) were “a bit scared” of contracting the infection. Only one-fifth (20.2%) of the hospital staff considered themselves to be well motivated to contribute their part to the prevention and control of COVID-19 while the majority, ninety nine (79.8%) reported that they were not well motivated to perform their roles. About half (52.0%) of the few participants who reported that they were well motivated to take care of COVID-19 patients owed it to personal sense of responsibility in taking care of the sick irrespective of the risks involved. About a quarter of them reported that they were motivated because they believed in their personal proficiencies and abilities to handle the disease while the remainder were motivated simply because they believed the disease could be prevented and controlled. The major reasons given by respondents for “lack of motivation” were inadequate facilities and equipment (including PPEs) and resources accounting for almost 50% of the reports Figure 2. Other reasons included poor political will by government, lack of insurance and incentives, and inadequate hazard allowances. Nurses and doctors generally had a better attitude score than the others (P < 0.001) Table 1.

**Figure 3:**
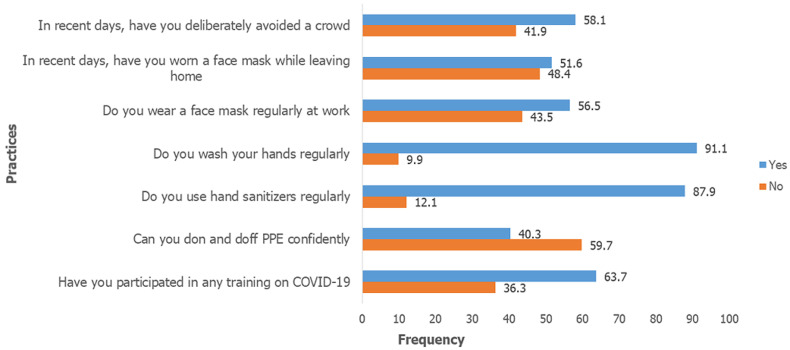
practice of hospital workers towards COVID-19

**Practice of participants towards COVID-19:** the mean practice score for all respondents was 4.55 + 1.64 with a range of 1.00 - 7.00 (Table 1). The practices of the hospital workers are illustrated in Figure 3. A good proportion (91.1%) practiced regular hand washing and use of sanitizers (87.9%). Only 43.5% wore a mask regularly at work while 63.7% had received a formal hospital-based training on COVID-19. Individuals with post-graduate level of education and those with graduate degree had a higher practice score compared with those with secondary level of education (p= 0.000). However, there was no difference in practice score between postgraduate and graduate level of education (p = 0.715). Also, medical doctors had a higher mean practice score than those in the “others” category with a marginally significant post hoc analysis test (p =0.047).

## Discussion

This was a study among workers of a tertiary government-owned hospital which was also a designated isolation centre for treatment of patients with COVID-19. Our study showed a generally high level of knowledge about COVID-19 in the hospital employees. High level of knowledge and awareness has been similarly reported in other studies on COVID-19 among heath care workers. A survey conducted in Henan, China among 1357 health care workers reported that 89% had sufficient knowledge about COVID-19 with doctors and nurses having higher knowledge score than the other health care workers [10]. This may be a reflection of the content and scope of their curriculum or continuous medical education they usually undergo. Higher knowledge about COVID-19 was noted in hospital workers who were less than 30yrs and who were unmarried as opposed to older married employees. The reason for this is not immediately clear but could probably be because the former engages more in social media and television programs considering that these were the most common sources of knowledge in our study. The use of internet and other social media avenues is generally known to be high among young people in Nigeria and other parts of the world with a significant impact on knowledge. School graduates were also found to have a significantly higher knowledge about COVID-19 than those with lower level of education. This positive association between knowledge of COVID-19 and level of education has been reported elsewhere [9]. It is not surprising that the level of knowledge was not affected by work experience. Since COVID-19 is a novel and evolving disease, much of what is known about it has only recently been discovered and more is still being unraveled. Knowledge is a prerequisite for establishing prevention beliefs, forming positive attitude and promoting positive behaviors. Majority (63.7%) of the participants in our study believed that COVID-19 would eventually be controlled by divine intervention. It is important to note that the confidence in most of these participants was predicated on religious grounds rather than in the health care system. Almost all the participants that took part in the study were Christians. Previous studies have reported a strong interplay between health and religious beliefs with some authors recommending that government consider input from religion in health care policy formulation [11]. Less than 40% of respondents believed that Nigeria would win the war against COVID-19. The principal reasons given by majority of respondents for this lack of optimism included poor political will, corruption and poor funding by government. It is feared that corruption could be a clog in the wheel of Nigeria´s response to this pandemic which could further worsen and overwhelm the already weak and fund-starved health systems in the country.

This study also reported low level of motivation and a high level of fear of contracting the virus or dying in a majority of respondents with over half of them attributing this to poor government funding, inadequate facilities, and lack of PPE. Poor remuneration as hazard pay and lack of health insurance packages also adversely affect staff motivation. There has been a clamour for an increase in the hazard allowances of Nigerian health workers in recent times. The need for the development of appropriate health insurance packages for health care workers has also come to the front burner particularly occasioned by the pandemic. The welfare of health care workers has often been relegated to the background. This is however, different from the experience in China where the frontline medical staff involved in management of the COVID-19 epidemic reported receiving material and psychological support and care, making them more motivated and increasing the confidence in their ability to defeat the virus [10,12]. Safety practical measures are required for preventive care among health care workers. In this study, regular hand washing and the use of hand sanitizers were commonly practiced among the majority of the participants. Hand hygiene has been widely adopted and accepted as a preventive measure against COVID-19 infection. Handwashing with soap and water helps to inactivate the virus. The virus is also said to be unable to survive when exposed to alcohol, the main ingredient in most hand sanitizers. The use of face masks was initially `not widely accepted due to controversies on whether it was beneficial to persons who were not directly in contact with ill persons. However, in line with the WHO recommendation on regular wearing of face masks in May 2020 [13], this has since been widely adopted. For those who manage very ill patients the use of surgical and N95 face masks have been recommended as part of the PPE and has been found to be effective in protecting against the disease. These safe practices are in agreement with the safety measures recommended in a US-based study [14].

Unavailability or inadequacy of PPE may have been a reason why a good proportion of our respondents were scared of contracting the disease. It has been suggested that in order to prevent contamination and risk of infection, health care workers should place high value on the practical use and correct removal of PPE especially in high risk environment [15]. A number of participants, particularly among the doctors and nurses, reported that they knew how to don and duff their PPE. This knowledge may have been acquired from participating in hospital-based seminars and workshops on COVID-19 which several of them also alluded to. It is however note-worthy that the attitude of these hospital workers was not commensurate with the high knowledge they exhibited. This relatively poor attitude was reported to have been influenced by factors such as perception of lack of political will and poor funding by government and relevant stakeholders, insufficient PPE and lack of staff motivation. This study has some limitations. Since the survey was conducted in only one facility, it may be difficult to generalize the findings. The sample size for the study was small. Furthermore, the convenient sampling method was used to recruit all participants who were available or who could be reached in the facility at the time of study. However, as a non-probability method, it could introduce some bias. Most of the participants in the study were young with a short working experience. This will also affect the extent of generalizability of the study. Another limitation of the study is reduced objectivity. The practices reported were based on participants´ responses which may be different from actual reality.

## Conclusion

In summary, the study has demonstrated a high level of knowledge of COVID-19 and fairly adequate safety prevention practices towards the disease among relatively young hospital staff of a tertiary hospital. However, their attitude was poor as several of them were not well motivated to work due to a number of factors including inadequate equipment and resources, lack of political will by government, lack of insurance and poor incentives and allowances for health care workers. In order to be able to successfully combat the present pandemic, all hands need to be on deck. There is an urgent need for the support of government and other stakeholders particularly in the aspects of increased funding of the health sector, health advocacy and implementation of favorable health policies. This will be necessary if the health care work force is to be adequately mobilized and motivated to work to curb this ravaging pandemic. There would subsequently be need for large scale prospective studies to examine the effect of specific interventions and policies on attitude and practices of health care workers.

### What is known about this topic

- COVID-19 caused by the novel SARS-CoV-2 virus presents as one of the most devastating outbreaks that the world has known in recent times;
- Health workers occupy a central role in the management of epidemics;
- Health care workers need to be knowledgeable about COVID-19 with the right attitude and practice in management of the outbreak.

### What this study adds

- The study showed a low level of motivation among hospital workers;
- The poor motivation was ascribed to inadequate provision of equipment and resources for work as well as fear of contracting the infection, and issues of incentives, allowances and insurance;
- Underscores the need to provide support and work-related equipment and resources for health care workers.
